# Instrumental Color Measurements Have Relationships to Fat Smearing in Fresh Sausage

**DOI:** 10.3390/foods12142813

**Published:** 2023-07-24

**Authors:** Jarrod Bumsted, Emily Ford, Amanda Blair, Keith Underwood, Stacy M. S. Zuelly

**Affiliations:** 1 Department of Animal Sciences, South Dakota State University, Brookings, SD 57007, USA; jarrod.bumsted@tyson.com (J.B.); amanda.blair@sdstate.edu (A.B.); keith.underwood@sdstate.edu (K.U.); 2 Department of Animal Sciences, Purdue University, West Lafayette, IN 47907, USA; eford21@purdue.edu

**Keywords:** fresh sausage, fat smearing, instrumental color, processed meat quality

## Abstract

Fat smearing, or poor fat particle definition, impacts the visual quality of sausage. However, objective methods of assessing fat smearing have not been identified. Therefore, the objective of this experiment was to determine the relationship between fat smearing and instrumental color analysis for fresh sausages to create a standard method for using instrumental color in fat smearing analysis. Meat blocks of pork (PK), beef (BF), and a mixture of pork and beef (P/B) were formed and processed at three different temperatures to create varying degrees of fat smearing. The average fat smearing score of each sausage was used to determine if a relationship existed with instrumental color measurements (CIE L*, a*, b*, and reflectance percentage at 580 nm and 630 nm) and color calculations. A correlation was observed for L* (R = −0.704) and the reflectance at 580 nm (R = −0.775) to PK fat smearing (*p* < 0.05). In P/B sausage, both reflectances at ratios between 630 nm and 580 nm were correlated to P/B fat smearing. No measurement or calculation was correlated with BF fat smearing (*p* > 0.05). Therefore, it is possible to use instrumental color analysis for the evaluation of fat smearing in pork and pork/beef blended sausage products, but not in beef sausage products.

## 1. Introduction

Sausage manufacturing processes involve grinding to reduce particle size, mixing to incorporate spices, and stuffing into casings. During manufacturing, fat smearing can occur, which gives the product a muddled appearance after stuffing and cooking. Smearing is when fat within the product loses its particle definition, and it is caused by a combination of unsaturated fatty acid composition and increasing sausage temperature [1]. Animal fats with high concentrations of unsaturated fatty acids have a lower melting point, causing a softer texture at colder temperatures than saturated fat [2,3]. Unsaturated fats, such as pork fat, are widely used because of the flavor they contribute to the finished sausage product. Consequently, the softness of unsaturated fat and the elevated temperatures caused by the mechanical action of sausage production can result in noticeable fat smearing that may decrease consumer acceptance and increase labor and processing costs [1].

Standard objective and subjective evaluations have the ability to measure product quality; however, there is no standardized analysis method available to evaluate fat smearing in fresh sausage products. Previous fat evaluations rely on human subjective analysis that can be highly variable or not available to all researchers [4]. However, several rapid, inexpensive technologies exist to evaluate meat color that could potentially replace subjective analysis and are nondestructive to the products being evaluated [5]. Hunter Lab values or CIE L, a, and b values, known as L*, a*, and b* are utilized by numerous researchers to measure meat color [6]. These measurements evaluate color as three components: L* = +light/−dark, a* = +red/−green, and b* = +yellow/−blue. These values can be reported as individual values or ratios of a*/b* to determine the difference in hue angle and saturation index [6]. In addition, reflectance ratios obtained from specific wavelengths are commonly used to determine surface color and changes in color over time [6]. However, the use of instrumental color analysis is largely focused on the lean meat color and has not been applied to the analysis of fat smearing.

Since fat smearing can drastically change the perception of a high-quality fresh sausage product, instant, noninvasive strategies may provide a detectable relationship between color and fat smearing. Our hypothesis was that instrumental color measurements have a relationship to observed fat smearing and can be used as a method for the analysis of fat smearing. The objective of this experiment was to determine the relationship between fat smearing and instrumental color analysis for fresh pork, beef, and blended sausage products to create a standard method for using instrumental color in fresh sausage analysis in order to quantify fat smearing.

## 2. Materials and Methods

### 2.1. Sample Preparation

Beef and pork trim were purchased and transported to the South Dakota State University Meat Laboratory, Brookings, SD, USA, and formulated into meat blocks consisting of 100% pork (PK), 100% beef (BF), and 50% pork + 50% beef blend (P/B) in triplicate batches (*n* = 9). Each batch was formulated to be 80% lean and 20% fat, and proximate analysis was conducted to verify the lean-to-fat ratio. Each batch was then divided into three equal sub-portions (*n* = 27). To create a distribution of fat smearing, each batch sub-portion was tempered to one of three temperature treatments: low temperature (−1.1 °C), medium temperature (4.4 °C), and high temperature (10 °C). Each batch was ground twice with a 4.76 mm grinding plate, mixed with 2 percent salt and 0.3 percent pepper, and stuffed into 5.1 cm diameter sausage casings. Each sausage was crust frozen and transversely bisected into slices, resulting in sausage patties (2.54 cm thick). Three patties were fabricated from the medial portion of each sausage. Two patties were packaged in PVC overwrap for visual and instrumental color analysis and one patty was vacuumed packaged, frozen, and stored at −20 °C for proximate analysis.

### 2.2. Fat Smearing Measurements

Visual analysis was conducted to determine the degree of fat smearing in each tray of patties by seven trained evaluators. Evaluators were trained using the standard scale created by Varnold et al. [7], and evaluators were determined proficient when able to have greater than 95% repeatability in scoring to the standard. One package from each sausage blend and temperature treatment (*n* = 27) was evaluated by utilizing a 15 cm anchored line scale to score each tray of patties. The anchors of the scale were 0 = no fat smearing and 15 = extreme fat smearing.

### 2.3. Instrumental Color Analysis

The same packages evaluated for fat smearing were placed in a cooler (3.3 °C) under fluorescent lighting. The instrumental color analysis included CIE L* (lightness; 0 = black, 100 = white), a* (redness/greenness; positive value = red, negative values = green), and b* (yellowness/blueness; positive values = yellow, negative values = blue). Instrumental color measurements were recorded in duplicate on the exposed cut surface of each patty, using a Minolta Chroma Meter CR-310 (Minolta Corp., Ramsey, NJ, USA) with a 50 mm diameter measuring area and a D65 illuminant. Reflectance percentages were measured for each patty at 580 nm and 630 nm, using a Hunter Mini Scan XE (Hunter Associates Laboratory, Inc., Model 45/0-L; Reston, VA, USA). All calculations for color parameters are outlined in AMSA [6]. Reflectance ratios were calculated for 630 nm − 580 nm and 630 nm/580 nm. Redness and discoloration were calculated using a*/b*. Chroma (saturation index) was calculated using the following formula:

Chroma = (a*^2^ + b*^2^)^1/2^ (1)

Finally, the hue angle was calculated using the following formula:

Hue angle = arctangent (b*/a*) (2)

### 2.4. Proximate Analysis

A chemical analysis of the sausage was conducted to determine the moisture and fat of each sample in duplicate. The samples were immersed in liquid nitrogen and subsequently powdered with a Waring commercial blender (Waring Products Division, New Hartford, CT, USA). Replicate 2 g samples were dried in tin foil pans at 100 °C (24 h), and percent moisture was calculated as the difference between the original weight and dried weight. Ashless, N-free filter paper was then wrapped over the samples and tin foil pan and extracted with petroleum ether in a side arm soxhlet (60 h) for ether extraction of lipid followed by drying at 101 °C for 24 h [8]. Crude fat was calculated as the difference between dried sample weight and extracted sample weight.

### 2.5. Statistical Analysis

This experiment was designed for the correlation and regression analysis of instrumental color measurements and calculations in relation to the visual analysis of fat smearing. Data were analyzed using the correlation (Proc Corr) and regression (Proc Reg) procedure of the SAS software package (SAS version 9.4; SAS Institute Inc., Cary, NC, USA, 2012). To determine the possibility of a multiple linear regression model, Proc Reg was utilized with the selection criteria of the greatest adjusted R^2^ and lowest Akaike information criterion (AIC) to determine the best-fit model. All correlations and regressions were determined significant at *p* < 0.05. To validate that fat content was consistent between replications, Proc GLM was used to ensure there were no differences found *p* > 0.05.

## 3. Results and Discussion

Proximate analysis confirmed that each sausage blend had no significant differences between the replicates (*p* > 0.05); thus, fat concertation would not vary among the samples to alter the fat smearing scores. Correlation coefficients (R) and regression coefficients (R^2^) for individual instrumental color measurements and calculations in relation to visual fat smearing are displayed in Table 1.

**Figure 1:**
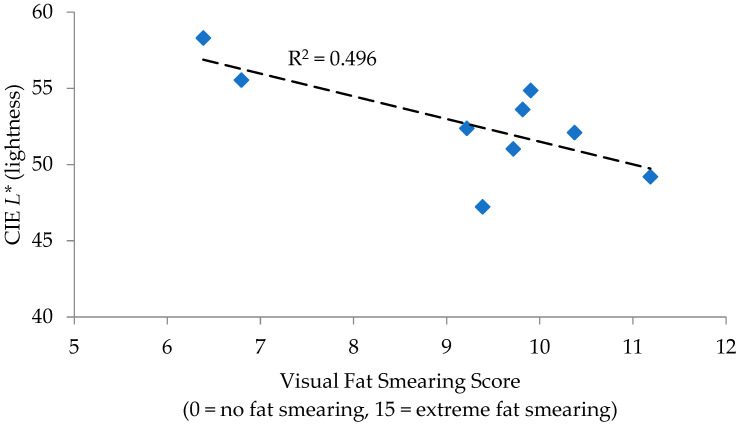
Regression analysis of CIE L* to visual fat smearing score for pork sausage.

**Figure 2:**
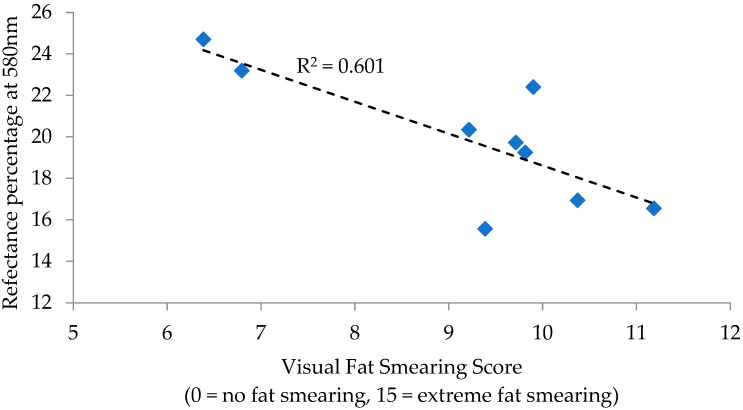
Regression analysis of the reflectance percentage at 580 nm to visual fat smearing score for pork sausage.

For PK sausage, correlations to fat smearing were observed for L* values (R = −0.704; *p* = 0.034) and the reflectance percentage at 580 nm (R = −0.775; *p* = 0.014), with remaining measurements and calculations lacking significance (*p* > 0.05). Regressions for these individual measurements determined L* values to have an R^2^ of 0.496 (Figure 1) and the reflectance percentage at 580 nm to have an R^2^ of 0.601 (Figure 2) in relation to fat smearing. L* values are a measurement of lightness (+light/−dark), while reflectance at 580 nm is generally used in the calculation of the reduction of metmyoglobin (brown in color) to oxymyoglobin (red in color). The similarities indicated that a darker, redder color is indicative of greater fat smearing.

For BF sausage, the correlation of instrumental color measurements and calculations lacked significance to fat smearing (Table 1; *p* > 0.05). However, a* values and the reflectance percentage at 580 nm were approaching significance (*p* = 0.078 and *p* = 0.080, respectively). This trend is similar to the findings in PK sausage that darker and/or redder values correlated to increased fat smearing. The smeared fat particles in the BF sausage may not have effectively altered to affect the color scores once smeared due to the increased red fibers containing a greater amount of myoglobin found in beef verse pork [8]. In addition, beef contains a large amount of saturated fatty acid within the muscle, meaning the fat is more solid or firm than unsaturated fatty acids at similar temperatures [9,10,11], indicating that fat smearing may be less severe in fresh beef sausage.

**Figure 3:**
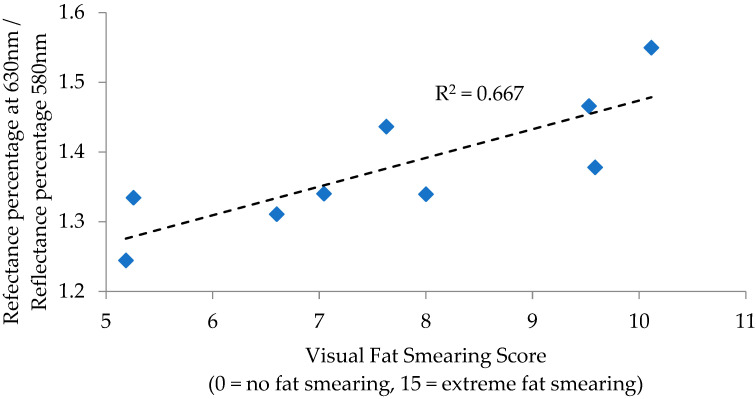
Regression analysis of the reflectance percentage at 630 nm/reflectance percentage at 580 nm to visual fat smearing score for pork and beef blended sausage.

**Figure 4:**
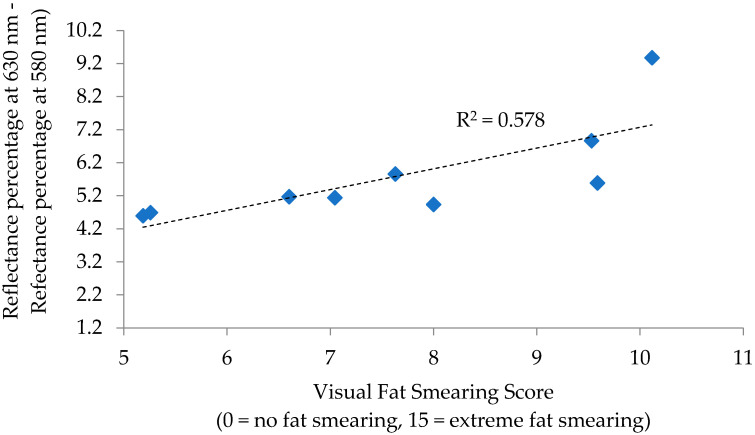
Regression analysis of reflectance percentage at 630 nm—reflectance percentage at 580 nm to visual fat smearing score for pork and beef blended sausage.

For P/B sausage, correlations to fat smearing were observed for the reflectance ratios of 630 nm/580 nm (R = 0.817; *p* = 0.007) and 630 nm − 580 nm (R = 0.760, *p* = 0.017), with remaining measurement and calculations lacking significance (*p* > 0.05). Regressions for these individual calculations determined 630 nm/580 nm to have an R^2^ of 0.667 (Figure 3) and 630 nm − 580 nm to have an R^2^ of 0.578 (Figure 4) in relation to fat smearing. These ratios can be used in fresh meat analysis as measurements of discoloration, as larger ratios indicate more redness due to either oxymyoglobin or deoxymyoblogin [6]. In this way, the findings in P/B sausage follow a similar logic as those found in the PK sausage, that redder colors relate to increased fat smearing.

According to Weiss et al. [1], as fat smearing increases, the fat particles are no longer well-defined in the surrounding protein matrix. Thus, as fat smearing increases, the overall color of the sausage should become darker in color due to the lack of white fat particle definition. The data collected from this analysis show that, in general, color measurements that indicate darker, redder values correspond with increased fat smearing, particularly in PK and P/B sausages.

Further analysis of the data analyzed the color measurements and calculations in a multiple linear regression using a selection criterion in SAS for the greatest R^2^ and lowest AIC to determine the best-fit model. The regression equations for each sausage type are displayed in Table 2. P/B had a significant prediction equation (*p* = 0.006), while BF had an equation approaching significance (*p* = 0.061), and PK was not significant (*p* = 0.118). Although some of these equations were significant and could generate R^2^ extremely close to 1.0, the number of parameters and calculations could be considered convoluted. This would require further analysis to determine a standardized equation, and, therefore, the individual measurements and/or calculations may offer a more practical application in the research setting to evaluate fat smearing.

## 4. Conclusions

Previous research has reported correlations between numerous instrumental color measurements and visual lean color scores [6]. Thus, utilizing CIE L*, a*, and b* values and/or calculation ratios of these instrumental color values offers the potential to be successful in the detection of fat smearing differences.

Differences in fat smearing predictors were found between the different sausage types. These data indicate that the most effective method for determining the degree of fat smearing in PK is L* and reflectance wavelength at 580 nm. There were no measurements that showed a correlation to fat smearing in BF, indicating a need for additional research in this area. Reflectance ratios were found to be significant predictors of fat smearing in P/B sausage. Overall, the use of instrumental color analysis to determine the amount of fat smearing in sausages is an effective method, with the trend throughout all sausage types being that redder, darker color measurements relate to increased fat smearing. However, the use of another methodology for color analysis, such as near-infrared color analysis would be useful in conjunction with the instrumental use of the Minolta and Hunter Lab devices.

## Figures and Tables

**Table 1:** Correlation and regression analysis of instrumental color (CIE L* (lightness), CIE a* (redness), and CIE b* (yellowness)), reflectance values, reflectance ratios, chroma, and hue angle of pork, beef, and mixed sausages to visual fat smearing scores.

	Sausage Type ^1^								
	PK			BF			P/B		
Measurement ^2^	R	R^2^	*p*-Value	R	R^2^	*p*-Value	R	R^2^	*p*-Value
L*	−0.704	0.496	0.034	−0.298	0.089	0.436	−0.009	<0.000	0.982
a*	0.388	0.151	0.302	0.615	0.378	0.078	0.312	0.097	0.413
b*	0.376	0.141	0.319	0.113	0.013	0.772	−0.048	0.002	0.903
580 nm ^3^	−0.775	0.601	0.014	−0.612	0.375	0.080	−0.193	0.037	0.619
630 nm ^3^	−0.539	0.291	0.135	0.359	0.129	0.342	0.338	0.114	0.374
630 nm/580 nm	0.419	0.176	0.262	0.514	0.264	0.157	0.817	0.667	0.007
630 nm − 580 nm	0.038	0.001	0.923	0.527	0.278	0.145	0.760	0.578	0.017
a*/b*	0.243	0.059	0.529	0.555	0.308	0.121	0.311	0.097	0.416
Chroma ^4^	0.487	0.237	0.184	0.577	0.333	0.104	0.078	0.006	0.842
Hue angle ^5^	−0.230	0.053	0.552	−0.561	0.315	0.116	−0.311	0.097	0.416

^1^ PK = 100% pork sausage, BF = 100% beef sausage, P/B = 50% pork and 50% beef sausage. ^2^ L* = CIE L* (lightness): 0 = black, 100 = white; a* = CIE a* (redness/greenness): positive = red, negative = green; b* = CIE b* (yellowness/blueness): positive = yellow, negative = blue; ^3^ reflectance percentage at 580 nm and 630 nm; ^4^ chroma (saturation index) = (a*^2^ + b*^2^)^1/2^; ^5^ hue angle = arctangent (b*/a*).

**Table 2:** Analysis of multiple linear regression models for the prediction of fat smearing using instrumental color measurements and calculations with the selection criteria of the greatest adjusted R^2^ and lowest Akaike information criterion (AIC) to determine the best-fit model.

Sausage Type ^1^	Multiple Linear Regression Equations ^2^	Adjusted R^2^	*p*-Value
PK	y = −1193.49 + L* (3.10) + a* (13.04) + 580 nm (−4.13) + 630 nm/580 nm (−33.88) + a*/b* (499.81) + Chroma (−4.53) + Hue angle (815.38)	0.991	0.188
BF	y = 121.09 + L* (−2.23) + b* (19.66) + 630 nm (4.54) + 630 nm/580 nm (18.91) + 630 nm − 580 nm (−3.80) + Chroma (−15.50) + Hue angle (−127.79)	0.993	0.061
P/B	y = 115.75 + L* (−1.77) + a* (−31.27) + b* (−49.25) + 580 nm (0.37) + 630 nm/580 nm (−28.41) + 630 nm − 580 nm (3.98) + Chroma (57.24)	0.999	0.006

^1^ PK = 100% pork sausage, BF = 100% beef sausage, P/B = 50% pork and 50% beef sausage. ^2^ L* = CIE L* (lightness): 0 = black, 100 = white; a* = CIE a* (redness/greenness): positive = red, negative = green; b* = CIE b* (yellowness/blueness): positive = yellow, negative = blue; reflectance percentage at 580 nm and 630 nm; chroma (saturation index) = (a*^2^ + b*^2^)^1/2^; hue angle = arctangent (b*/a*).

## Data Availability

Data are contained within the article.
